# P-1810. Implementation of the Core Elements of Hospital Antibiotic Stewardship Programs and the Priorities for Hospital Core Element Implementation in Long-term Acute Care Hospitals Reporting to the National Healthcare Safety Network, 2014 and 2023

**DOI:** 10.1093/ofid/ofae631.1973

**Published:** 2025-01-29

**Authors:** Katryna Gouin, Sarah Kabbani, Erin O’Leary, Arjun Srinivasan, Melinda M Neuhauser

**Affiliations:** Centers for Disease Control and Prevention (CDC), New York, New York; Centers for Disease Control and Prevention, Atlanta, Georgia; Centers for Disease Control and Prevention, Atlanta, Georgia; Centers for Disease Control and Prevention, Atlanta, Georgia; Division of Healthcare Quality Promotion, Centers for Disease Control and Prevention,, Atlanta, GA

## Abstract

**Background:**

The Centers for Disease Control and Prevention (CDC) defines the 7 *Core Elements of Hospital Antibiotic Stewardship Programs* as a framework for antibiotic stewardship implementation. In 2022, CDC released 6 *Priorities for Hospital Core Element Implementation* to enhance the quality and impact of existing antibiotic stewardship programs. Acute care hospitals reported 97% uptake of the 7 Core Elements and 10% uptake of the 6 Priorities in 2022 as per reporting to the National Healthcare Safety Network (NHSN) Annual Hospital Survey. However, stewardship implementation in long-term acute care (LTAC) hospitals has not been widely assessed. The objective of this analysis was to assess the uptake of the 7 Core Elements and 6 Priorities in LTAC facilities in 2023 and compare Core Elements uptake to 2014.

**Table 1.**
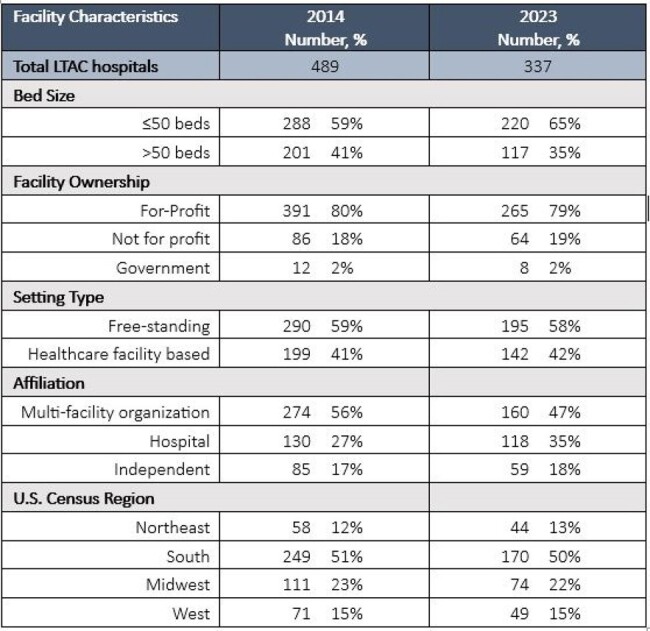
Facility Characteristics of Long-Term Acute Care (LTAC) Hospitals Reporting to the National Healthcare Safety Network Annual Facility Survey for LTAC, 2014 and 2023

**Methods:**

We used the NHSN Annual Facility Surveys for LTAC hospitals (4/1/2024 data pull) for survey years 2014 and 2023 to characterize facility characteristics and self-reported responses to antibiotic stewardship program survey questions. We mapped stewardship survey questions to corresponding Core Elements and Priorities to describe the reported uptake of the 7 Core Elements and 6 Priorities.

**Figure 1.**
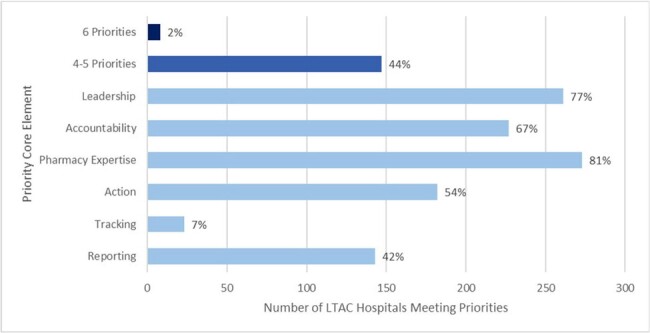
Reported Implementation of the Priorities for Hospital Core Element Implementation by Priority in Long-Term Acute Care (LTAC) Hospitals (N=337), 2023

**Results:**

In 2014 and 2023, 489 and 337 LTAC hospitals completed the annual survey. In 2023, most LTAC facilities were for-profit (79%), free-standing (58%), part of multi-facility organizations (47%), and in the South (50%) (Table 1). From 2014 to 2023, the number of facilities reporting uptake of all 7 Core Elements in LTAC hospitals increased from 60% to 98%. In 2023, 2% of LTAC hospitals reported meeting the 6 Priorities and 44% reported meeting 4-5 Priorities. The highest reported uptake of Priorities across LTAC hospitals was Pharmacy Expertise (81%) and Leadership (77%). The lowest reported uptake of Priorities was Tracking (7%) and Reporting (42%) (Figure 1).

**Conclusion:**

LTAC hospitals reported uptake of the Core Elements of Antibiotic Stewardship increased from 2014 to 2023, on par with the increase acute care hospitals reported for Core Elements implementation. Engagement with LTAC hospital leadership and leveraging pharmacy expertise are important to support antibiotic stewardship Priorities, especially tracking & reporting of antibiotic use.

**Disclosures:**

**All Authors**: No reported disclosures

